# How Individualized Niches Arise: Defining Mechanisms of Niche Construction, Niche Choice, and Niche Conformance

**DOI:** 10.1093/biosci/biac023

**Published:** 2022-05-11

**Authors:** Rose Trappes, Behzad Nematipour, Marie I Kaiser, Ulrich Krohs, Koen J van Benthem, Ulrich R Ernst, Jürgen Gadau, Peter Korsten, Joachim Kurtz, Holger Schielzeth, Tim Schmoll, Elina Takola

**Affiliations:** Department of Sociology, Anthropology and Philosophy, University of Exeter, Exeter, England, United Kingdom; Department of Philosophy, Bielefeld University, Bielefeld, Germany; Center for Philosophy of Science, University of Münster, Münster, Germany; Department of Philosophy, Bielefeld University, Bielefeld, Germany; Department of Philosophy, University of Münster, Münster, Germany; Department of Theoretical Biology, Bielefeld University, Bielefeld, Germany, and with the Groningen Institute for Evolutionary Life Sciences, University of Groningen, Groningen, The Netherlands; Universität Hohenheim, Apicultural State Institute (Landesanstalt für Bienenkunde), Stuttgart, Germany; Institute for Evolution and Biodiversity, University of Münster, in Münster, Germany; Institute for Evolution and Biodiversity, University of Münster, in Münster, Germany; Department of Animal Behaviour, Bielefeld University, Bielefeld, Germany; Institute for Evolution and Biodiversity, University of Münster, Münster, Germany; Population Ecology Group, Institute of Ecology and Evolution, Friedrich Schiller University Jena, Jena, Germany; Department of Evolutionary Biology, Bielefeld University, Bielefeld, Germany; Population Ecology Group, Institute of Ecology and Evolution, Friedrich Schiller University Jena, Jena, Germany

**Keywords:** niche construction, individualized niche, individual differences, phenotypic plasticity, habitat choice

## Abstract

Organisms interact with their environments in various ways. We present a conceptual framework that distinguishes three mechanisms of organism–environment interaction. We call these *NC^3^ mechanisms*: niche construction, in which individuals make changes to the environment; niche choice, in which individuals select an environment; and niche conformance, in which individuals adjust their phenotypes in response to the environment. Each of these individual-level mechanisms affects an individual's phenotype–environment match, its fitness, and its individualized niche, defined in terms of the environmental conditions under which the individual can survive and reproduce. Our framework identifies how individuals alter the selective regimes that they and other organisms experience. It also places clear emphasis on individual differences and construes niche construction and other processes as evolved mechanisms. The NC^3^ mechanism framework therefore helps to integrate population-level and individual-level research.

Organisms change their environment: Beavers  build dams, birds build nests, and earthworms alter soil structure. These organisms thereby modify their living conditions and the selective regime under which they live. These modificatory actions are often called *niche construction* (Lewontin 1983, Laland et al. 2015).

We see two problems with common understandings of niche construction. First, there is often confusion between the individual and the population level. When individual organisms modify their environments, they first affect their own *individualized* niches. Only subsequently do they alter the population niche and thereby population-level ecological and evolutionary processes. Recognizing that individuals modify their own niches also helps to understand the importance of individual differences in niche construction.

Second, research on niche construction is often focused on the way organisms make changes to their environments. But organisms can alter their individualized niches in a number of other ways. For instance, both phenotypic change and relocation also affect the niche of an individual. We distinguish three mechanisms by which organisms can alter their niches: niche choice, niche conformance, and niche construction. We call these *niche-altering mechanisms* or *NC^3^ mechanisms*, the cubed indicating that each mechanism forms a different dimension along which an individual can modify its niche. Giving each mechanism its own label and clarifying the distinguishing features promotes empirical research, because it makes it easier to identify and study how organisms alter their niches.

By identifying different types of niche-altering mechanisms, we provide a unified framework of organism–environment interactions that integrates individual-level and population-level perspectives. We argue that the different ways organisms alter their niches are evolved causal mechanisms that play a role in evolutionary processes. These insights derive from our work within the interdisciplinary research consortium “A Novel Synthesis of Individualisation across Behaviour, Ecology and Evolution: Niche Choice, Niche Conformance and Niche Construction (NC^3^)” (www.uni-bielefeld.de/biologie/crc212). We focus mostly on animals, but we see our framework as applicable to plants and other taxa, too.

The present article is structured as follows. We first point out that niche construction is carried out by individuals and that there can be individual differences in niche construction. We then subdivide the umbrella concept of niche construction into three distinct mechanisms through which individuals interact with their environments, the NC^3^ mechanisms. All three mechanisms alter the phenotype–environment match and the individual's fitness, thereby determining the niche of an individual. Finally, we discuss how our approach integrates individual-level and population-level research, with a focus on the role of individual activities in evolutionary changes.

## Individual differences in niche construction

Niche construction is sometimes characterized as the process by which an organism “influences its own evolution” (Lewontin and Levins 1985, p. 106) by changing the environment. This requires clarification. On the one hand, it is individual organisms (alone or in groups) that alter the environment. On the other, it is populations, not individuals, that evolve in response to this altered environment.

Niche construction theorists highlight that “niche construction is typically expressed by individual organisms, but natural selection is a process that operates within populations” (Odling-Smee et al. 2003, pp. 41–42). In other words, niche construction involves individuals changing the environment and thereby altering population-level selection pressures.

Niche construction therefore emphasizes the role of individual organisms in ecological and evolutionary processes. In this way, the concept of niche construction differs from that of the extended phenotype, which is focused on the environmental effects of genes but ignores the intervening causal steps at the level of the organism (Dawkins 1982, 2004, Hunter 2009, Wells 2015, Laland et al. 2016).

Recognizing individuals as the agents of niche construction invites consideration of an often overlooked aspect of niche construction: individual differences. Not all individuals in a population are the same, and different individuals can construct niches in different ways. These individual differences in niche construction are important because they can have evolutionary consequences (Saltz and Nuzhdin 2014).

Being clear that we are talking about *individual* activities invites us to focus on a particular sort of niche—namely, the *individualized* niche. The idea that individuals have their own, individualized niches has been proposed following the recognition of ecological specialization within populations (Roughgarden 1972, Bolnick et al. 2003, Dall et al. 2012, Violle et al. 2012, Müller et al. 2020). This extends to individualized social niches, where the focus is on an individual's relations to conspecifics (Bergmüller and Taborsky 2010, Montiglio et al. 2013, Saltz et al. 2016).

For instance, queens of the California harvester ant (*Pogonomyrmex californicus*) vary in their colony-founding behavior, either tolerating other queens or killing them (Rissing et al. 2000, Clark and Fewell 2014). This individual difference leads to fundamentally different social structures with either multiple unrelated queens or a single queen (Overson et al. 2014, Overson et al. 2016). It seems that colony density, the degree of territoriality or aggression, and resource availability (Haney and Fewell 2018) are crucial components of the selective environment that favors either one or the other type of niche construction.

We summarize our definitions of species and individualized niches in box 1 and explain them in detail later in the text. Briefly, we consider it important to look not only at differences in the environment (Grinnell 1917) or trophic positions (Elton 1927). We therefore use the distinction between the set of viable conditions and the concrete conditions in the actual habitat, as was captured by Hutchinson's (1957) concepts of the fundamental and realized niche.

**Box 1. The ecological niche of an individual.**

The ecological niche of a species is the set of environmental conditions under which it exists and can maintain itself (implying nonnegative population growth rates in the long run; Hutchinson 1957).

We define the fundamental individualized niche as the range of environmental conditions under which a specific individual with a given set of traits could possibly live and reproduce.

We define the realized individualized niche as the environmental conditions under which a specific individual does actually live and reproduce. The realized niche is therefore the subset of the fundamental niche realized in the actual environment in which an individual lives.

We emphasize the dynamic nature of individualized niches. A fundamental individualized niche can change through the individual's activities that alter its physiological or behavioral phenotypes or developmental pathways. A realized individualized niche can change through activities that alter the environment or that alter with which (parts of the) environment the individual interacts.

Hutchinson (1957) focused on the conditions under which a population as a whole persists; he therefore paid little attention to the fact that some individuals might be better off under conditions that are less favorable for others. In contrast, the individualized niche concerns how the requirements and dispositions of an individual relate to its environment, including the social environment. Individuals that differ from each other will, in the very same environment, experience different effects of environmental conditions, consume different resources, have different interactions with conspecifics, and so on, and these interactions can have different fitness effects (Krüger et al. 2021 [https://doi.org/10.32942/osf.io/7h5xq; preprint: not peer reviewed], Takola and Schielzeth 2022).

Individual organisms engage in many sorts of activities altering their individualized niches. Most of the classical examples of niche construction involve organisms actively making changes to their environments, rather than, for instance, changing the environmental conditions they live in by relocation. This makes sense, because we tend to intuitively understand niche construction in analogy to human construction of artifacts such as buildings or roads (Archetti 2015).

However, many niche construction theorists understand niche construction more broadly. Odling-Smee, Laland, and Feldman (2003) included relocating as an instance of niche construction. Others include phenotypic alteration that changes how an environment is experienced as a kind of niche construction (Lewontin and Levins 1985, Chiu and Gilbert 2015, Sultan 2015, Chiu 2019). For instance, Aaby and Ramsey (2022) delineated three kinds of niche construction: constitutive construction occurring via phenotypic change, relational construction via a change in organism–environment relationships, and external construction via changes to the physical environment.

The use of the term *niche construction* to cover such diverse phenomena is potentially confusing, especially given that paradigmatic examples continue to revolve around making changes to the environment (Okasha 2005, Archetti 2015). We therefore suggest restricting the concept of niche construction to what we see as its intuitive scope: organisms making changes to their environment. To refer to other ways in which individuals interact with their environment and thereby alter their niches, we propose two additional terms: *Niche choice* occurs when individuals select an environment, and *niche conformance* occurs when individuals alter their phenotype in response to the environment (see also Edelaar and Bolnick 2019). Collectively, we refer to niche construction, niche choice, and niche conformance as *niche-altering mechanisms*, or *NC^3^ mechanisms*.

Our framework offers similar distinctions to those of niche construction theorists such as Aaby and Ramsey (2022) but has the advantage of restricting *niche construction* to its intuitive scope. It is also distinctive in focusing explicitly on individual activities and their effects on individualized niches.

## Introducing the NC^3^ mechanisms

NC^3^ mechanisms consist of entities and activities that are spatially, temporally, and hierarchically organized in specific ways and produce a phenomenon. This accords with definitions of mechanisms put forward in the philosophy of science (Bechtel and Richardson 1993, Glennan 1996, 2017, Machamer et al. 2000, Craver and Darden 2013). The activities that individuals carry out lead to a specific outcome: a change in the phenotype–environment match, in the individual's fitness, and in its individualized niche. Referring to niche construction, niche choice, and niche conformance as mechanisms highlights that we seek to understand the causal process of *how* individuals interact with their environment and thereby change match, fitness, and individualized niches.

NC^3^ mechanisms share a general structure. Usually, they are organized around a *focal individual*. The focal individual, sometimes in cooperation with other individuals, is engaged in a *focal activity*. Activities are what organisms do. The philosophical term *activity* (like *behavior*) also includes changes in which an organism is not moving or exerting large amounts of energy, such as resting or going to sleep. Activities can involve one or more entities. These entities can be active, performing the activity, or they can be passive, having the activity done to them (Machamer et al. 2000, Illari and Williamson 2013, Kaiser 2018). For example, parasites are actively involved in the activity of infecting, whereas their hosts have a passive role in this activity (although hosts may respond in turn by, for example, raising an immune response to the infecting parasites). Similarly, beavers are actively involved in the activity of cutting trees, whereas trees are passively involved in this activity (although tree species may, in turn, respond evolutionarily to the cutting activity; Bailey et al. 2004). For the NC^3^ mechanisms, the focal individual is actively involved in the focal activities.

**Figure 1.**
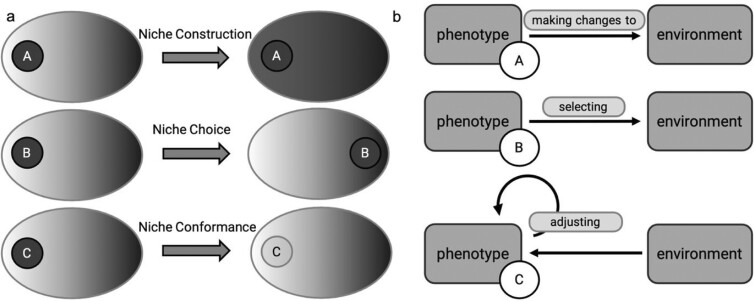
NC^3^ mechanisms. (a) Three individuals, A, B and C are in the same environment. Each individual uses a different NC^3^ mechanism, resulting in a change of its phenotype–environment match. (b) Each NC^3^ mechanism involves a different focal activity. Focal individuals A, B, and C can make changes to the environment, select the environment, or adjust the phenotype in response to the environment. (b) is modified from Kaiser and Trappes (2022).

The different NC^3^ mechanisms are discerned by the respective focal activity of the individual. In the case of niche construction, the focal activity is to make changes to the environment; in niche choice, the focal activity is to select (parts of the) environment with which the focal individual interacts; and in niche conformance, the focal activity is to adjust the phenotype in response to the environment (figure 1). These focal activities are quite abstract; in descriptions of concrete NC^3^ mechanisms, they are replaced by more specific activities involving the focal individual, its internal states, other organisms, and abiotic factors (Kaiser and Trappes 2022). In the example of harvester ants, for instance, the niche construction activities involve aggressive or tolerant behavioral interactions with other queens, as well as certain colony-founding tasks.

The outcome of NC^3^ mechanisms—that is, the phenomenon they produce—can also be characterized on a general level. NC^3^ mechanisms change the match between the focal individual's phenotype and its environment, as well as the individual's fitness, which is understood as the number of offspring produced or its contribution to the gene pool of future generations as we explain later in the article. Consequently, NC^3^ mechanisms also change an individual's niche, taken preliminarily as the set of (social and nonsocial) environmental parameter values under which it survives and reproduces (box 1). This is why we refer to the three focal activities (making changes to the environment, selecting an environment, and adjusting the phenotype in response to the environment) as *niche-altering activities*.

It is important to note that the NC^3^ mechanisms refer not to a choice of, conformance to, or construction of the niche but, rather, to a choice of, conformance to, or construction of the *environment*, which, in turn, affects the niche. When it comes to individualized social niches, the NC^3^ mechanisms specify three different ways by which individuals can become specialized in their social niches, a phenomenon referred to as *social niche specialization* (Bergmüller and Taborsky 2010, Montiglio et al. 2013, McCune et al. 2018).

Box 2 summarizes the general structure of NC^3^ mechanisms. The general structure, together with the distinction of the three focal activities, gives rise to definitions for the NC^3^ mechanisms.

**Box 2. The focal activities of NC^3^ mechanisms.**

In NC^3^ mechanisms, a focal individual is actively involved in a *focal activity*, which leads to a change of the phenotype–environment match, a fitness change, and a change of the individualized niche.

Niche construction is the mechanism by which an individual *makes changes to its environment*, resulting in a change of the individual's phenotype–environment match, fitness and individualized niche.

Niche choice is the mechanism by which an individual *selects an environment*, resulting in a change of the individual's phenotype–environment match, fitness and individualized niche.

Niche conformance is the mechanism by which an individual *adjusts its phenotype*, resulting in a change of the individual's phenotype–environment match, fitness and individualized niche.

The NC^3^ mechanisms do not have to occur independently. Often, two or even all three mechanisms will occur in sequence (e.g., during different life stages of an individual) or even simultaneously. For instance, a bird building a nest is certainly a case of niche construction, but individuals ready to breed often need to relocate to a suitable habitat and choose a tree in which to build their nest, so that niche choice precedes niche construction.

In addition, the very general nature of the NC^3^ mechanism definitions in combination with the fact that mechanisms can act in concert means that classification may be ambiguous in some cases. For instance, an organism making changes to the environment (niche construction) typically involves certain behavioral or other phenotypic changes, as occurs in niche conformance. Empirically differentiating niche construction and niche conformance is particularly challenging when it comes to social niches. It is difficult to detect whether a focal individual changes its social behavior first, which then causes a change in the behavior of its conspecifics, or whether it is the other way around. Similarly, choosing a specific social group (niche choice) will also induce changes in the social dynamics of that group, as occurs in niche construction.

An illustrative example of such ambiguity in separating the NC^3^ mechanisms is posed by the cooperatively breeding cichlid *Neolamprologus pulcher*. In the early postnatal period, individuals already develop integrated behavioral and dispersal phenotypes (or behavioral syndromes; Sih et al. 2004), which predispose them to either staying as a helper in their natal breeding group or leaving to become a breeder elsewhere (Bergmüller and Taborsky 2007, Fischer et al. 2017). In this example, niche conformance and niche choice go hand in hand. The emergence of different behavioral phenotypes may also influence the dynamics of the social group, adding an element of social niche construction. Each case must therefore be considered carefully in light of the NC^3^ mechanisms at play.

## Three types of niche-altering activities

In this section, we analyze the three focal activities in more detail and provide some examples. Later, we tackle the other elements of the definitions: the way individual activities lead to changes in phenotype–environment match and fitness and their effects on individualized niches.

### Rebuilding the environment: Niche construction

Mechanisms of niche construction are characterized by the focal individual actively making changes to its environment. The classic examples of niche construction cited earlier—beavers building dams, birds building nests, and worms altering soil structure—involve focal individuals, often in pairs or groups, altering the properties of their abiotic environment (for many more classic examples, see Odling-Smee et al. 2003). However, individuals can also make changes to their biotic or social environment through interactions with organisms from other species or with conspecifics.

A special case, *social niche construction*, occurs when individuals change their social environment, especially by altering the way conspecifics behave by interacting with them. For example, in cooperatively breeding meerkats (*Suricata suricatta*), dominant females show increased aggression toward pregnant subordinate females, resulting in their temporary eviction from the social group. Being evicted induces severe stress in the subordinates, promoting spontaneous miscarriages. In this way, dominant females shape their social environment, suppressing their competitors’ reproduction, and thereby monopolizing reproduction in the group (Young et al. 2006).

An interaction with other organisms or the abiotic environment must satisfy two conditions to count as the focal activity in niche construction. First, there must be a modification of the environment and not just a change in *which* environment or environmental factors the individual relates to (which would be niche choice). Second, the focal individual must have an active role (see above). This excludes changes in the environment in which an individual is only passively involved, such as changes in a nonhuman organism's environment produced by human activities such as farming or anthropogenic climate change. On the other hand, activities such as defecation or trampling may well be considered niche construction, because the focal individual is actively engaging in activities that change its environment, as long as the focal individual's phenotype–environment match and fitness also change as a result. These two conditions are captured in our definition (box 2) by the phrase *makes changes to its environment*.

### Selecting the environment: Niche choice

The second NC^3^ mechanism is niche choice, in which the focal individual selects the environment or parts of the environment with which it interacts. In cases of niche choice, individuals change how they interact with different parts of the environment rather than changing their phenotype (niche conformance) or making changes to the environment (niche construction).

A paradigmatic type of niche choice is an individual moving to a different habitat, known as *habitat choice* (Edelaar et al. 2008, Edelaar and Bolnick 2019). Sometimes, this is a temporary or context-dependent choice. For instance, in azure sand grasshoppers (*Sphingonotus azurescens*), darker individuals prefer darker underground, whereas individuals with lighter colors prefer lighter underground. Interestingly, manipulation of individuals’ color (with dark or light paint) led to changes in preferences, such that dark-painted individuals preferred darker underground, independent of their original, natural coloration (Camacho et al. 2020). An example of a more permanent niche choice is territory establishment. For instance, movement data from juvenile black grouse (*Tetrao tetrix*) showed that, following natal dispersal during the first year, most individuals remained in the areas in which they spent their first winter (Caizergues and Ellison 2002).

Note that, in addition, competition between individuals for limited high-quality habitat can result in phenotype–environment correlations if certain individuals are forced into lower quality habitat (rather than selecting this on the basis of their preference in the absence of competition; Fokkema et al. 2021). Although such competition-induced phenotype–environment correlation can be viewed as a fundamental ecological phenomenon in its own right (Fokkema et al. 2021), the level of competition for certain habitats can also be seen as an integral part of the ecological and social context in which individuals are selecting their environment through niche choice.

Niche choice does not necessarily involve physical relocation but can include selective interactions with parts of the environment, especially through choice of resources or social groups. An illustrative example of individual differences in the choice of the social environment is provided by cliff swallows (*Petrochelidon pyrrhonota*); individuals have heritable preferences for breeding in smaller or larger groups (Brown and Brown 2000). Another example are the California harvester ants referred to earlier: Queens choose whether or not to found a nest together with other queens.

In most cases of niche choice, the focal individual changes its location, resource use, or interactions with its environment. But what about an individual choosing to not change anything? We think that these cases should qualify as niche choice if the individual was able to explore different options of changing its relationship with the environment. Even if this exploration does not result in a change of the individual–environment relationship, it does involve individuals actively approving their present environments. It might be difficult to discern such cases empirically from cases in which an individual does *not* make a choice (because it does not explore different environmental options). Nevertheless, there is a conceptual delineation of the cases; it is then an interesting empirical question whether a particular individual that has maintained the same relationship with its environment has engaged in choice behavior.

### Adjusting the phenotype: Niche conformance

The third NC^3^ mechanism is niche conformance, which involves focal individuals changing their phenotypes in response to environmental conditions. Niche conformance involves phenotypic plasticity, the capacity of an organism to develop distinct phenotypes in response to environmental variation (Pigliucci 2001, 2005). Accordingly, the focal activity of adjusting the phenotype, which characterizes niche conformance (box 2), can also be described as adjusting the phenotype in response to certain environmental conditions. But niche conformance involves more than just the focal activity.

Although similar to phenotypic plasticity, niche conformance also includes how phenotypic adjustment leads to changes in the phenotype–environment match, fitness, and the individualized niche of the focal individual. In particular, by stressing interindividual variation in organisms’ plastic responses, so-called individual-by-environment interactions (Nussey et al. 2007), it directly leads to the expectation that the resulting niches are individualized. Therefore, niche conformance amends classic examples of phenotypic plasticity (see Pigliucci 2001) with their consequences for phenotype–environment match, fitness, and individualized niches.

Niche conformance may accommodate any kind of trait (morphological, physiological, behavioral, or life history) and can be applied to irreversible developmental changes during an individual's lifetime (nonlabile traits), as well as to reversible changes in response to the current environment (labile traits). An example for a nonlabile, morphological trait is the expression of inducible defenses such as spines and helmets by water fleas (e.g., *Daphnia cucullata*) during development in response to anticipated variation in predation risk (Laforsch and Tollrian 2004). An example for a labile life-history trait is the timing of reproduction in many temperate bird species, such as the great tit (*Parus major*); individual females track between-year variation in spring temperatures in order to match supply and demand in nestling food (Charmantier et al. 2008). Another example is provided by *Macrostomum hystrix*, a flatworm, in which individuals are facultative selfers that reproduce through outcrossing when a mating partner is available but self-fertilize if no partner has been available for a certain amount of time (Ramm et al. 2012).

## NC^3^ mechanisms affect phenotype–environment match, fitness, and the individualized niche

In this section we discuss the outcome of the NC^3^ mechanisms. We begin with the changes to phenotype-­environment match and fitness. We then introduce in detail the concept of an individualized niche and discuss how the NC^3^ mechanisms affect individualized niches.

### Phenotype–environment match and fitness

The NC^3^ mechanisms bring about changes in the phenotype–environment match and in fitness. The notion of *match* that we use is evaluative, referring to how well an organism's phenotype matches to its environment. Match is often measured by reproductive success. Match can also be assessed using other measures that are positively correlated with fitness, such as food uptake rate, body condition, efficiency of locomotion, and effectiveness of coloration (e.g., camouflage, warning colors). Such measures may serve as fitness proxies, because an improvement of factors such as body condition or effective mimicry will often increase an organism's fitness (Krohs 2022).

A niche altering mechanism will usually result in a change of absolute fitness. Specifically, the altered match between the organism and its environment will, in general, lead to more or fewer surviving offspring (which is, incidentally, one reason reproductive success is often used to measure match). It is the specific activity of the focal individual that changes its reproductive success. Changes in absolute fitness will often also affect relative fitness (i.e., fitness relative to other individuals in the population), and relative fitness drives evolutionary change. Whether a particular NC^3^ mechanism leads to a change in relative fitness depends crucially on the activities of other individuals in the population. NC^3^ mechanisms in our definition are therefore not immediately linked to evolutionary change.

Nevertheless, we expect that evolved NC^3^ mechanisms will tend to increase focal individuals’ (absolute and relative) fitness (Odling-Smee et al. 2003, p. 48). Because most NC^3^ mechanisms are costly, they would likely not have been maintained if they did not also provide a benefit. This assumption is reflected in many empirical and theoretical studies in which the adaptive value of NC^3^ mechanisms has been investigated (Callahan et al. 2014, Nicolaus and Edelaar 2018, Crowley et al. 2019; for a contrasting assessment, see Davidson et al. 2011).

An example that illustrates the adaptive value of both niche choice and niche conformance is provided by the variable coloration of grasshoppers (Rowell 1972). A green-brown color polymorphism is widespread across a large number of species (Schielzeth 2020 [https://doi.org/10.1101/2020.03.31.016915; preprint: not peer reviewed]) and must therefore be maintained by balancing selection favoring alternative phenotypes. Interestingly, some species are able to conform their body color to the background of the habitat (Rowell 1972, Dearn 1990), whereas others search out microhabitats to achieve a phenotype–environment match (Heinze et al. 2021). Color variation in grasshoppers is also variable within color morphs. Grasshoppers are able to adjust their body coloration both to improve crypsis and thereby reduce predation (Baños-Villalba et al. 2018, Camacho et al. 2020) and to improve temperature regulation (Valverde and Schielzeth 2015, Köhler and Schielzeth 2020). Crypsis has direct fitness consequences, and attaining sufficiently high body temperature is generally important to ectotherms and likely affects fitness by allowing for more extended periods of activity. Grasshoppers therefore use at least two of the NC^3^ mechanisms to improve the phenotype–environment match and fitness, both on the coarse level of alternative color morphs and fine-grained individual variation.

It might seem natural to define the NC^3^ mechanisms in a way that reflects the expectation of fitness increases, such that NC^3^ mechanisms not just change but improve match and fitness of the focal individual. However, we decided not to restrict the definitions of the NC^3^ mechanisms to improvements in match and fitness for two reasons.

First, requiring that NC^3^ mechanisms increase fitness would mean that a specific mechanism would not qualify as an NC^3^ mechanism when, because of some intervening factor, the overall outcome is disadvantageous or neutral for the focal individual. Second, even constant or lower absolute fitness could be beneficial in evolutionary terms if relative fitness increases. This would be the case if, for instance, conspecifics suffer more than the focal individual from the operation of an NC^3^ mechanism (as in spiteful behavior; Hamilton 1970). Similarly, it is theoretically expected that the benefit of a costly social behavior such as cooperation depends not only on an individual's behavior but also on the variation for the behavior in the rest of the population (McNamara and Leimar 2010). The fitness effects of NC^3^ mechanisms therefore depend on the specific conditions under which they take place—in particular, on social conditions and activities of other individuals in a population.

It is for such reasons that we believe NC^3^ mechanisms should also allow for instances of an activity that do not increase individual fitness or other measures of match. Such cases can be particularly relevant to study, because they can illuminate why individuals engage in apparently nonadaptive activities—for example, in evolutionary traps (Schlaepfer et al. 2002). An additional benefit of our neutral definition of NC^3^ mechanisms is that a mechanism can be identified prior to studying its fitness effects in detail, thereby facilitating communication and research about these mechanisms across diverse study systems.

### Individualized niches

So far, we have addressed individual activities and their consequences for match and fitness. The last element of the NC^3^ mechanisms is their effect on individualized niches. We understand the individualized niche as the individual-level counterpart of the Hutchinsonian population niche (Hutchinson 1957, Holt 2009, Krüger et al. 2021 [doi.org/10.32942/osf.io/7h5xq; preprint: not peer reviewed], Takola and Schielzeth 2022). Redefining Hutchinson's concept at the individual level requires several modifications.

First, Hutchinson defines the niche in terms of the environmental conditions under which a population could persist indefinitely, a condition that needs some modification to be transferable to the case of individuals with necessarily limited life spans. We define the limits of the individualized niche in terms of individual realized fitness equal to or greater than what is necessary for the individual to replace itself in future generations (i.e., each individual leaving the equivalent of one reproducing offspring). In addition, in the context of the NC^3^ mechanisms, we are interested in how exposure to different environmental parameters can change fitness above the minimum required for replacement. We therefore need to consider gradual fitness values for different subsections of the niche, as Hutchinson proposed for population niches (Hutchinson 1957). Therefore, the individualized niche is the mapping, or function, of fitness over sets of environmental parameters.

Second, to accommodate changes of niche relations within a lifetime, the quality of match needs to be evaluated with respect to a given situation or short period of time. Realized fitness, being a lifetime outcome, is not the appropriate parameter. Instead, fitness proxies such as individual (ontogenetic) growth rate, territory possession, mating rates and fertilization success can indicate how suitable a combination of niche parameter values is for an individual at a given time.

Third, we need to distinguish between the fundamental niche and realized niche at the individual level (box 1). We define a fundamental individualized niche as the environmental conditions under which an individual could possibly live and reproduce. An individualized realization of a niche is in turn the subset or region of the fundamental niche realized by the environmental conditions under which the individual actually survives and reproduces. We call this a *realized* individualized niche. We should note, however, that the distinction between fundamental and realized individualized niche does not exactly mirror Hutchinson's concepts. For instance, fundamental individualized niches might include some level of competition between individuals. In contrast, the fundamental population niche excludes competition between populations. Some authors proposed to use the term *potential individualized niche* instead of *fundamental individualized niche* to emphasize the difference from Hutchinson's fundamental niche (Takola and Schielzeth 2022).

There are two important consequences of recognizing individualized niches in addition to population niches. First, individuals have an additional set of niche dimensions based on social interactions, as is highlighted in the concept of the social niche (Saltz et al. 2016, Takola and Schielzeth 2022). Second, recognizing individualized niches allows individual differences to inform both theoretical and empirical research in ecology and evolution (Bolnick et al. 2003, Dall et al. 2012, Violle et al. 2012). In particular, as we elaborate in the next section, it allows us to clarify how individuals affect their niches through niche-altering mechanisms.

### NC^3^ mechanisms alter individualized niches

**Figure 2.**
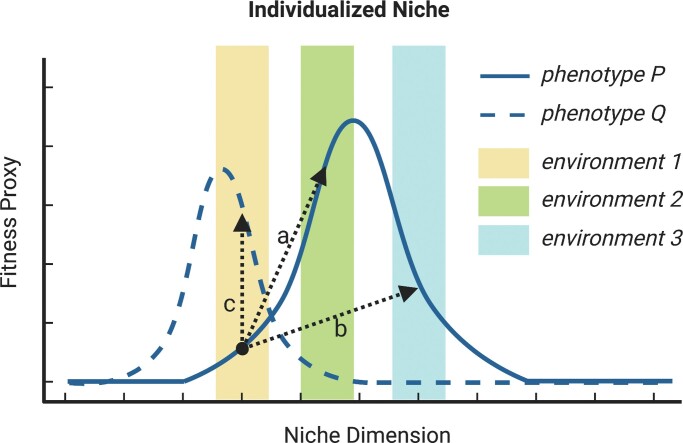
NC^3^ mechanisms produce changes in the individualized niche. Simplified individualized niches, showing two possible phenotypes and their values for fitness or match (e.g., growth rate, fertilization success, performance) along a single niche dimension (e.g., temperature, food abundance). (a) Niche construction: An individual with phenotype *P* in environment 1 makes changes to its environment so that it becomes environment 2, thereby increasing the individual's fitness. (b) Niche choice: An individual with phenotype *P* in environment 1 selects environment 3, thereby increasing its fitness. (c) Niche conformance: An individual with phenotype *P* in environment 1 adjusts to phenotype *Q*, thereby increasing its fitness within environment 1.

Individuals conform to, choose, or construct the environment, and these activities result in changes of individualized social or nonsocial niches (figure 2).

Niche construction produces a change in the values of environmental factors such as temperature, humidity, resource availability, social group size, or the presence of conspecifics with a given social rank. Niche construction therefore affects which set of niche parameters is realized in the environment of the individual. As such, niche construction changes the realized individualized niche.

Because niche construction changes the selective regime, it might also lead to an evolutionary response. If phenotypic variation has a heritable basis, then natural selection may change the phenotypes of future individuals; phenotypic change through natural selection could in turn alter the fundamental individualized niches of future individuals. For example, most bird species construct elaborate nests in which eggs are incubated and chicks raised until fledging (Hansell 2000, Mainwaring et al. 2014). Variation in nest construction among breeding individuals or pairs influences the nest's insulating properties and thereby the microenvironment experienced by the parents and their offspring (Mainwaring 2017). It may also affect mate choice and parental investment (Jelínek et al. 2016). Nest construction can in turn facilitate natural selection on phenotypes, potentially leading to evolutionary changes in both nest building and temperature tolerance (again, assuming such phenotypic variation is heritable).

The consequences of niche choice for the realized individualized niche are the same as for niche construction, but they are brought about by selecting a different (social or nonsocial) environment instead of modifying the existing environment (figure 2). Individual differences in choice driven by factors such as physiology, morphology, or social dynamics can lead to individuals realizing different niches. For instance, in the invasive racer goby (*Babka gymnotrachelus*), intraspecific interactions drive dispersal, because subordinate individuals tend to move greater distances in search of resources and less hostile social environments (Grabowska et al. 2019).

Niche conformance changes the individual's phenotype. A different phenotype means that the organism can, for instance, tolerate different conditions or exploit different resources. This, in turn, means that the individual will have changed its fitness value in the absence of any change in the environmental conditions. In this case, the fundamental individualized niche changes because of a change in the shape of the fitness function across the niche dimensions. In addition, the individual may, given its new phenotype, be able to survive and reproduce under new sets of environmental conditions. In this case, the fundamental individualized niche changes because new niche parameter values are included in the niche.

Interestingly, a change in the realized individualized niche can eventuate in a change in the fundamental individualized niche. This can happen when niche choice or construction is followed by niche conformance. For example, individual oystercatchers specializing on either soft or hard prey types grow different bill shapes that facilitate efficient processing of their specific prey of choice (van de Pol et al. 2010). In this case, birds specializing on a particular resource (niche choice) consequently adjust their phenotype (niche conformance). They thereby alter not only their realized individualized niche but also—by altering their performance on different resources and, therefore, the fitness function over the resources—their fundamental individualized niche.

## NC^3^ mechanisms and the synthesis of research on different levels

Our conception of the NC^3^ mechanisms as ecological and evolutionary mechanisms combines aspects of individual-centered research with considerations about their impact at the population level. This combination allows for a full acknowledgment of NC^3^ mechanisms in their own right as well as in their role as evolutionary mechanisms. We further assume that NC^3^ mechanisms have an evolutionary history and are to be conceived and analyzed as adaptations to environmental conditions in the past (which may or may not correspond to current conditions).

Our focus on individual activities, individual-level mechanisms and individualized niches extends the initiative from biologists who investigate niche construction in the wide sense. The importance of individual behavior for evolution was even stressed by Mayr (1963, p. 604), a notorious apologist of population thinking. Niche construction theory has brought a much stronger focus on analyzing evolutionary processes at the level of individual activities, acknowledging individuals’ status as subjects or agents that have an active role in evolution (Odling-Smee et al. 2003, Chiu and Gilbert 2015, Sultan 2015).

Our focus on mechanisms comprised of a focal individual and its focal activity stresses the importance of individuals’ activities (including developmental processes) as modulators of the selective regime under which individuals live. When the constructed or chosen environment is inherited, individual activities can also affect the realized individualized niches of future generations, leading to longer-term evolutionary effects (Pontarotti 2020). Our view also allows for novel activities arising by developmental processes that do not have a history of adaptation and may still be relevant to phenotype–environment match (West-Eberhard 2003).

Nevertheless, we do not propose a general answer to the question of whether organisms are evolutionary agents rather than mere objects of evolution. Instead, our framework invites solving this question empirically. This is in contrast to the at times staunchly held positions in the debate about the modern synthesis and extended evolutionary synthesis (Wray et al. 2014, Laland et al. 2014). Whether an individual's activity drives evolution in a new direction does not depend on subscribing to a theoretical framework. It is rather something that can and should be studied empirically.

Scrutinizing the NC^3^ mechanisms shows that this empirical study needs to rely on research that acknowledges and investigates individual differences in behavior, physiology, and morphology. In particular, it remains an important challenge to link the results of individual-based research with investigations on the population level, such as the study of population genetics.

## Conclusions

Organisms modify their niches in a number of different ways. We distinguish three different mechanisms by which individuals alter their environments and thereby their niches: niche construction, niche choice, and niche conformance. The three mechanisms are distinguished by the focal activity performed by the focal individual. In niche construction, the individual makes changes to the environment; in niche choice, it selects an environment; and in niche conformance, it adjusts its phenotype in response to the environment. All of the NC^3^ mechanisms change individuals’ phenotype–environment match and their fitness. Because many examples of NC^3^ mechanisms have evolved and persisted through natural selection, we expect them to generally improve phenotype–environment match and fitness, as has indeed been shown in several of the examples we have cited.

One interesting feature of NC^3^ mechanisms is the way they alter individualized niches, including social niches. Niche construction and niche choice affect the realized niche, altering which options of the fundamental individualized niche are realized in the environment the individual experiences. In contrast, niche conformance affects the fundamental niche, either by affecting the individual's performance in a given range of conditions or by changing the conditions under which the individual can survive and reproduce. Because they are individual-level mechanisms, NC^3^ mechanisms highlight the potential for individual differences to affect how organisms alter their niches.

Our framework recognizes that individuals can be agents of evolution, affecting the selective pressures to which they are exposed and the resources and information available for generating new phenotypic variation. It therefore describes the importance of individualized research in a field that traditionally takes a population perspective. In addition, it provides new material for understanding the evolution of niche-altering individual activities through natural selection.

## Supplementary Material

biac023_Supplemental_File

## Author Biographical

Rose Trappes, Behzad Nematipour, Marie I. Kaiser and Ulrich Krohs share first authorship. Rose Trappes (r.g.trappes@exeter.ac.uk) is affiliated with Egenis, the Exeter Centre for the Study of the Life Sciences, in the Department of Sociology, Anthropology and Philosophy at the University of Exeter, in Exeter, England, in the United Kingdom. RT and Marie I. Kaiser are affiliated with the Department of Philosophy at Bielefeld University, in Bielefeld, Germany. Behzad Nematipour is affiliated with the Center for Philosophy of Science at the University of Münster, in Münster, Germany. Ulrich Krohs is affiliated with the Department of Philosophy at the University of Münster, in Münster, Germany. Koen J. van Benthem is affiliated with the Department of Theoretical Biology at Bielefeld University, in Bielefeld, Germany, and with the Groningen Institute for Evolutionary Life Sciences at University of Groningen, in Groningen, The Netherlands. Ulrich R. Ernst is affiliated with Universität Hohenheim, Apicultural State Institute (Landesanstalt für Bienenkunde), in Stuttgart, Germany. URE, Jürgen Gadau, and Joachim Kurtz are affiliated with the Institute for Evolution and Biodiversity at the University of Münster, in Münster, Germany. Peter Korsten is affiliated with the Department of Animal Behaviour at Bielefeld University, in Bielefeld, Germany. Tim Schmoll is affiliated with the Department of Evolutionary Biology at Bielefeld University, in Bielefeld, Germany. Holger Schielzeth and Elina Takola are affiliated with the Population Ecology Group, in the Institute of Ecology and Evolution at Friedrich Schiller University Jena, in Jena, Germany. For information on author contributions, please see the author contribution statement in the supplementary materials.
