# Supplementary material for: How Individualized Niches Arise: Defining Mechanisms of Niche Construction, Niche Choice, and Niche Conformance
Source: Bioscience. 2022 May 11;72(6):538–48. doi: 10.1093/biosci/biac023 (PMC9169896; doi:10.1093/biosci/biac023)
Supplement: biac023_Supplemental_File [file biac023_supplemental_file.docx]

How Individualized Niches Arise: Defining Mechanisms of Niche Choice, Niche Conformance and Niche Construction

**Author Contribution Statement**

*Conceiving the theoretical framework*

All authors

*Creating the initial draft and performing revisions of the manuscript*

Rose Trappes, Behzad Nematipour, Marie I. Kaiser & Ulrich Krohs

*Developing Figure 1a*

Peter Korsten and Rose Trappes

*Developing Figure 1b*

Marie I. Kaiser and Rose Trappes

*Developing Figure 2*

Rose Trappes, Holger Schielzeth, Uli Ernst

*Contributing examples and helping to revise the text and figures*

Koen J. van Benthem, Ulrich R. Ernst, Jürgen Gadau, Peter Korsten, Joachim Kurtz, Holger Schielzeth, Tim Schmoll, Elina Takola

*Approving the manuscript for submission*

All authors
